# Implementation of a Decision Support System to Enhance Movement Proficiency Assessment in Sport

**DOI:** 10.3390/jfmk10010086

**Published:** 2025-03-05

**Authors:** Xavier Schelling, Enrique Alonso-Perez-Chao, Sam Robertson

**Affiliations:** 1 Institute for Health and Sport (iHeS), Victoria University, Melbourne, VIC 8001, Australia; sam.robertson@vu.edu.au; 2 Department of Physical Activity and Sports Science, University Alfonso X el Sabio, 28691 Villanueva de la Cañada, Spain; eperezch@uax.es; 3 Department of Physiotherapy, Faculty of Medicine, Health and Sports, European University of Madrid, 28001 Villaviciosa de Odón, Spain

**Keywords:** subjective assessment, reliability, feedback, single-leg squat, subjective tests

## Abstract

**Background/Objectives**: This study aimed to determine the relationships between seven descriptors of movement proficiency used by an expert to grade an athlete’s single-leg squat and the overall subjective ‘grade’ and the ability to predict a ‘grade’ based on the descriptors. A secondary aim was to determine the relationships between biomechanical data, the expert-defined descriptors, and the subjective ‘grade’ and its ability to predict the descriptors’ presence and the overall ‘grade’. **Methods**: Single-leg squats in 55 male athletes were graded using expert evaluation, synchronized video, biomechanical data, and decision tree and logistic regression analysis. **Results**: The model that most accurately predicted ‘grade’ (94.7%) was a decision tree with the descriptors as inputs. The model with biomechanical data for the descriptor ‘foot’ was the most accurate one (96.3%), followed by ‘lumbar’ and ‘depth’ (85.2%), ‘knee’ (81.2%), ‘pelvis/hip’ (71.7%), and ‘trunk’ (62.3%). These accuracies followed similar order to the intra-rater agreement: ‘foot’ (0.789), ‘lumbar’ (0.776), ‘knee’ (0.725), ‘depth’ (0.682), ‘pelvis/hip’ (0.662), and ‘trunk’ (0.637), indicating that ‘trunk’, ‘pelvis/hip’, and ‘depth’ are potentially the hardest descriptors to assess by the expert. **Conclusions**: The models developed in this study demonstrate that subjective perceptions can be somewhat accurately explained through a small number of biomechanical indicators. The results of this study support the notion that human movement evaluations should consider both subjective and objective assessments in a complementary manner to accurately evaluate an athlete’s movement proficiency.

## 1. Introduction

Subjective quality assessments are commonplace in sport, including areas such as talent identification, skill acquisition, or judging performance [1,2,3]. They are expertise based and time and cost efficient but with the caveat of potentially suffering from human bias and/or poor reliability [3,4]. The subjective assessment of movement quality in athletes is a common evaluation in sport. These evaluations are usually part of most rehabilitation processes [5,6], as well as an indicator of the ‘readiness’ in healthy athletes [7]. An assumption of these types of assessments is that there is an association between movement quality and risk of injury and/or athletic performance [8] and that the athlete’s movement patterns can be optimized in terms of proficiency or to provide a competitive edge [9]. Compared to objective methods, subjective assessments generally show lower reliability and higher variability due to factors such as assessor experience or contextual influences. Although objective techniques, like motion capture and force plate analysis, offer quantifiable and repeatable measurements, they also present limitations, including high costs and reduced ecological validity [10].

Movement quality often includes a visual examination of standard movements, such as single and double leg squats, or landing, jumping, and cutting actions, whereby a sum of scores informs a recommendation around the status of the athlete [6,9,11]. Experts typically evaluate movement proficiency (MP) using descriptors, such as alignment control, joint stability, and movement fluency, which are assessed through visual inspection or specific scoring systems [12]. Nevertheless, there is little agreement as to which movement tasks can expose patterns that could lead to injury [8]. There is some controversy on the validity of these scores for injury or athletic performance prediction [13,14] and on their level of reliability, especially when comparing the results between evaluators [12]. However, while predicting injuries and athletic performance from movement features is challenging, as there are multiple contributing factors [8], there are many studies that have examined movements using kinematic and kinetic measurements seeking to identify such features [15,16]. Kinematic data are typically collected through motion capture systems, which use high-speed cameras and reflective markers to track joint angles, velocities, and accelerations [17]. Additionally, inertial measurement units (IMUs) have become increasingly popular due to their portability and ability to capture movement outside of laboratory settings. On the other hand, kinetic data are obtained from force plates or instrumented treadmills, enabling movement analysis in both controlled and field settings [18].

As new data types are added, it becomes more difficult for the practitioner to be able to consider them all in their evaluations. This is due at least partially to the characteristics of human decision making [19], which are conditioned by the available information (sometimes insufficient and fallible and others precise but unattainable), the available time to make the decision (a few minutes or several days), and the cognitive limitations of the decision maker (information processing and biases) [20]. Further, the reliability of human judgment has been shown to be sometimes inconsistent or biased, as evidenced in injury prediction [21], team sport officials [22], talent identification [23], artistic sport judges [24], wine quality [25], or academic grades [26], where practitioners, consciously or unconsciously, displayed bias towards specific decisions or were unreliable.

Thus, with more and better objective data becoming available [9], the aggregation of subjective assessments combined with objective data in an automated way may improve the accuracy, time efficiency, and reliability of practitioner decisions to conform a hybrid decision-making process with potentially higher efficiency and better performance [2,27].

The primary objective of this study was to explore the relationships between multiple descriptors [7] of MP used by an expert to assess athletes and the overall subjective grades, as well as the ability of these descriptors to predict the assigned grade. Secondly, we aimed to analyze the relationships between kinematic data collected during the quality assessments, the expert-defined descriptors, and the subjective ‘grades’, as well as the capacity of kinematic data to predict both the presence of specific descriptors and the overall grade.

## 2. Materials and Methods

### 2.1. Data

Subjective MP grades along with kinetic and kinematic data were collected from 55 male athletes with an average age of 23.4 years old (18–40, 4.6 SD). A total of 176 single-leg squats with hands on hips were subjectively graded for movement proficiency by a senior physiotherapist with more than 20 years of experience in professional sport. Kinetic and kinematic data were collected simultaneously. The athletes were instructed to perform 10 s of self-paced single-leg half squats focusing on ‘keeping good balance and form and not touching the ground with the free leg’. The athlete had two 10 s trials for each ‘side’ (left and right). Only the subjectively considered best activity of the two 10 s trials was stored in an athlete management system. The research’s protocols fulfilled the provisions of the Declaration of Helsinki [28] and were approved by Victoria University’s ethical committee (HRE 20–204).

#### 2.1.1. Subjective Grades

**Figure 1:**
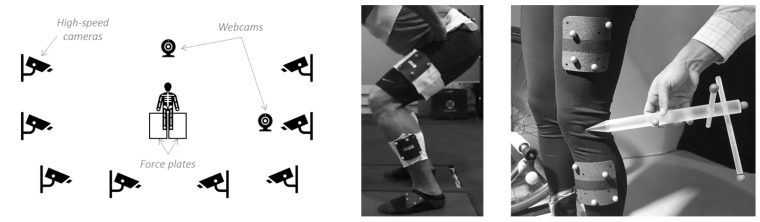
Schematic of the data collection setup featuring 2 force plates, 2 webcams, and 8 high-speed cameras (**left**), an example of marker cluster placements (**middle**), and a stylus for digitization (**right**). Photographs reproduced with permission from Innovative Sports Training Inc.

A synchronized two-camera recording system (LifeCam Cinema 720p HD Webcam, Microsoft, Redmond, WA, USA), managed by The Motion Monitor xGen 2.0 software (Innovative Sports Training Inc., Chicago, IL, USA), with frontal and sagittal views (Figure 1), was used to capture each activity and to visualize and ‘grade’ them a posteriori. The selection between the two 10 s trials was based on the expert’s overall impression of balance, depth, and form. If both trials were indistinguishable, the second trial was selected. On a 10 s self-paced continuous single-leg squats, a subject can perform between two and seven squats. Only the best squat based on squat depth, knee control, and pelvis/hip control was graded. The number of the selected squats was also recorded. Before giving the overall ‘grade’ to an activity, the expert reviewed a set of binary descriptors (correct or yes = 1/incorrect or no = 0) to evaluate what were considered key aspects of movement proficiency. There were 7 descriptors: foot, knee, pelvis/hip, lumbar, trunk, depth, and balance. For each of the seven binary descriptors, the expert used specific criteria to determine a correct or incorrect rating. For instance, ‘foot’ was rated as correct if the athlete maintained a stable, neutral position (no excessive pronation, supination, or toe out). ‘Knee’ involved assessing whether the knee tracked over the foot with minimal valgus/varus deviation. ‘Pelvis/hip’ focused on frontal plane stability (e.g., minimal contralateral hip drop) and neutral pelvic tilt. ‘Lumbar’ and ‘trunk’ examined the athlete’s ability to maintain an upright posture without excessive flexion/extension or lateral tilt. ‘Depth’ was considered adequate if the athlete reached the target squat depth (approximately 60° knee flexion) without compensations. Lastly, ‘balance’ was judged on the athlete’s ability to remain stable on one leg without excessive sway. Then, an overall proficiency ‘grade’ from 1 to 5 was given, 1 being ‘very poor’, 2 ‘poor’, 3 ‘fair’, 4 ‘good’, and 5 ‘excellent’. Table 1 is an example of a decision table for ‘grade’. Note that ‘grade’ is separate from being the sum of the descriptors.

#### 2.1.2. Biomechanical Data Collection

Eight high-speed cameras (Vicon Vero 1.2) with a sampling rate of 100 frames per second, controlled by the manufacturer’s software Tracker v3.20.2.0 (Vicon Motion Systems Ltd., Centennial, CO, USA), and two 40 × 60 centimeter force plates (Kistler 9260AA6, Winterthur, Switzerland) sampling at 1000 Hz were integrated into The Motion Monitor xGen software v3.7.0 (Innovative Sports Training, Inc., Chicago, IL, USA) (Figure 1). Eight clusters of reflective markers (B&L Engineering, Pinsco, Inc., Santa Ana, CA, USA) were located on the athlete’s feet (2), lower legs (2), thighs (2), sacrum (1), and upper back (1). ASIS (2), PSIS (2), C7/T1 (1), T12/L1 (1), L5/S1 (1), knees (2), ankles (2), and 2nd distal phalanx (2) were then digitized by a senior physiotherapist using the stylus provided by The Motion Monitor [29]. The capturing space and the force plates were calibrated as specified by the manufacturers before starting each subject’s recordings. A phase-zero Butterworth low-pass filter at 10 Hz was applied to the raw continuous kinematic data [30].

### 2.2. Statistical Analysis

All data were coded and analyzed in SAS Enterprise Guide (EG) version 7.15 and Enterprise Miner (EM) version 14.1 (SAS Institute Inc., Cary, NC, USA) analytical packages.

#### 2.2.1. Data Preparation

Subjective grades (1 to 5), descriptors (1, 0), and squat numbers did not need data manipulation. An algorithm to detect troughs and peaks was developed in EG7.15 to identify and number each squat from the continuous biomechanical data. The algorithm was applied to the knee flexion data and its derivatives (i.e., angular velocity). Average, standard deviation, maximum, minimum, range of degrees of motion, and value at max knee flexion of ankles, knees, hips, pelvis/hip, and trunk on frontal, sagittal, and transversal planes were calculated (Table 2). Total, antero-posterior, and mediolateral sway path, sway velocity, and the center of mass were extracted from the raw kinetic data. Grades, descriptors, and biomechanical data were joined on the subject, date, ‘side’, and squat number.

#### 2.2.2. Data Exploration

Distribution analysis of the subjective grades and the qualitative descriptors, as well as frequency plots showing the prevalence of each descriptor in each ‘grade’ and descriptive statistics (mean, standard deviation, max, min, range, and value at max knee flexion [31]) of the kinematic and kinetic data, were undertaken. Left and right single-leg squats were analyzed together (176 observations total), and ‘side’ was set as the classification variable. To determine the level of association between features, correlational and clustering analyses were undertaken for all kinetic and kinematic variables through variable selection and variable clustering nodes in EM14.1.

#### 2.2.3. Intra-Rater Agreement

In order to validate the reliability of the ‘grades’ and the descriptors, the expert was asked to re-assess 25% of the total sample, representing 44 single-leg squats. Cohen’s weighted kappa for ordinal response (‘grade’) [32] and Cohen’s unweighted kappa for nominal response (descriptors) were undertaken [33]. Kappa statistics (weighted and unweighted) were interpreted as <0.01 ‘poor agreement’, 0.01–0.20 ‘slight agreement’, 0.21–0.40 ‘fair agreement’, 0.41–0.60 ‘moderate agreement’, 0.61–0.80 ‘substantial agreement’, and 0.81–1.00 ‘almost perfect agreement’ [34].

#### 2.2.4. Data Sampling and Modification

For modeling purposes, various aspects of the biomechanical data required filtering outliers, where values beyond +/− 2 SD from the mean were replaced by missing values. Data was then partitioned (70% training and 30% validation, or 123 and 53 observations, respectively). Since the target variable (grade) was a class variable, a stratified partitioning was performed. Missing values were estimated and replaced by a tree method, analyzing each input as a target, and the remaining input and rejected variables were used as predictors [35]. For the tree imputation method, the leaf size was set at 5, the maximum branch at 2, the maximum depth at 6, the number of rules at 6, and the number of surrogate rules at 2. A dummy variable was generated for every imputed variable, indicating if the value for the variable in that observation was imputed or not. These indicators were used in subsequent modeling. Multiple data transformations on several biomechanical variables were performed using for the models the transformations that had the best chi-squared test for the target variable. The number of bins to use when performing optimal binning transformations was set at 4. This allowed for the discretization of some variables that balanced model fit and complexity [36].

#### 2.2.5. Modeling

For modeling purposes, four different approaches were taken: (1) grades as the target and descriptors only as the inputs, (2) grades as the target and kinetic and kinematic variables only as the inputs, (3) grades as the target and descriptors, kinetic, and kinematic variables as the inputs, and (4) the seven descriptors as the targets and kinetic and kinematic variables as the inputs. For each of the approaches, multiple models were developed in EM14.1 to determine the extent to which each of the features explained the target variable: ordinal logistic regressions (ORLs) with different variable selection methods and an optimized decision tree (DT) before and after replacing the missing values. In order to provide an understanding of any discrepancy between the model and the expert’s opinion, to ensure transparency, to facilitate expert validation, and to reduce the risk of unchecked biases in the predictive process, only easier-to-understand white-box models were developed in this analysis [4,37]. For each approach, a model assessment was performed to select the best predictive model based on their misclassification rates on the validation data.

General settings for the ordinal logistic regressions included setting ‘grade’ or each of the descriptors (foot, knee, pelvis/hip, lumbar, trunk, balance, and depth) as the target variable. In the case of ‘grade’, an ordinal variable, 5 (‘excellent’), was set as the reference category. When not the target, the binary qualitative descriptors as well as all kinetic and kinematic data were set as input variables. ‘Side’ (left, right) was set as the classification variable. The main effects for each input variable as well as their cross-effect with ‘side’ were invoked. The cumulative logit model was selected with Fisher’s scoring as the optimization technique and the misclassification rate as the assessment of the model fit. Multiple Bernoulli was the used error function. Stepwise, Forward, and Backward selection processes were performed, and the model with the best C statistic was the selected one. The significance levels to enter and stay in the model were set at *p* < 0.05. Confidence levels were set at 95%.

The Entropy method adjusted with ordinal distances was used to evaluate candidate splitting rules and to search for the best one when ‘grade’ (an ordinal variable) was the target variable. The ProbChisq method, which uses the *p*-value of the Pearson chi-square statistic for the target versus the branch node, was used when each of the seven binary descriptors was the target variable. The significance level (*p*-value) for the worth of a candidate splitting rule for the ProbChisq method was set at 0.05. For the Entropy method, the minimum gain threshold was also set at 0.05. Splitting variables could not be repeatedly used in splitting rules that apply to descendant nodes. The minimum number of train observations that a categorical value must have before the category can be used in a split search was set at 3. The minimum number of training observations that were allowed in a leaf node was set at 1. The misclassification rate was used as the assessment method to select a subtree from the fully grown/maximal tree for each possible number of leaves. Bonferroni adjustment to the *p*-values before the split was set. A maximal tree was also developed on each of the 4 analytical approaches as an exploratory exercise. This application allowed for a descriptive and visual analysis to be discussed with the senior physiotherapist in relation to her thought process when assessing movement proficiency.

## 3. Results

**Figure 2:**
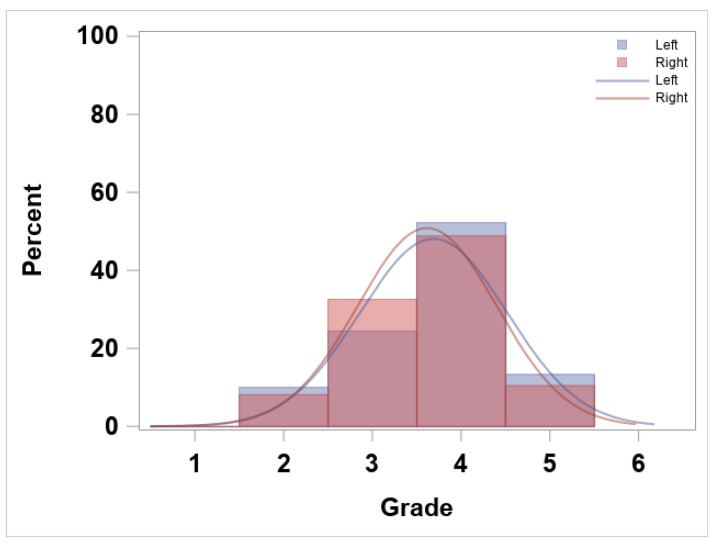
‘Grade’ frequency (%) and smoothed normal distribution plot by ‘side’ (**left** and **right**).

A total of 176 observations (90 left and 86 right) were analyzed. ‘Grade’ frequency distribution by ‘side’ is shown in Figure 2. The most common grades for both the left and right single-leg squats cluster around 3 (fair) and 4 (good), indicating that most athletes achieved moderate to good proficiency. There were no observations for ‘grade’ 1 (very poor), and very few squats were rated as 5 (excellent), suggesting that extreme performance levels were relatively rare. Overall, the grade distributions for the left and right legs are similar, with their histograms and fitted curves both peaking between 3 and 4, although the exact proportions of each grade differ slightly between sides.

**Figure 3:**
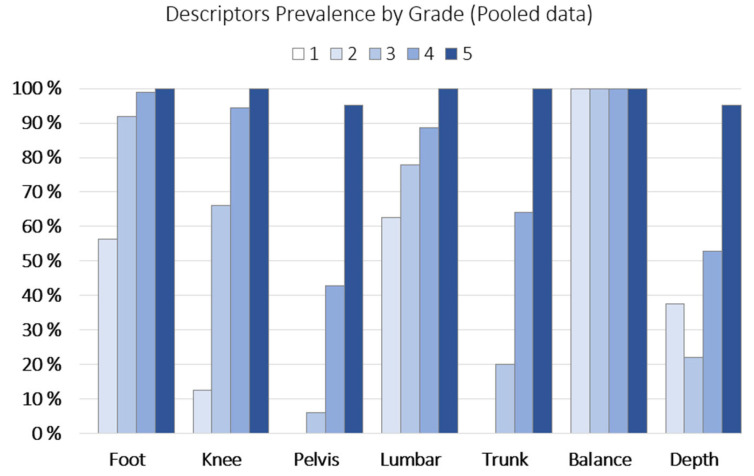
Descriptor prevalence by ’grade’ (pooled data).

The frequency (%) of descriptors present per grade is shown in Figure 3.

**Figure 4:**
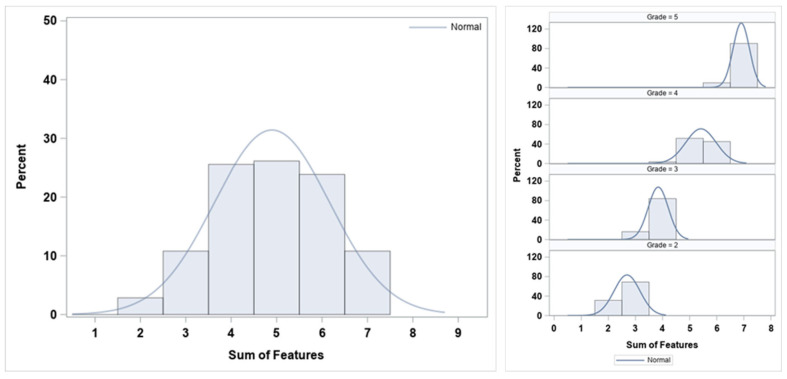
Overall (**left**) and by ‘grade’ (**right**) frequency distribution (%) of the sum of descriptors and their respective smoothed lines for normal density distributions.

The overall distribution of the sum of descriptors and by ‘grade’ is shown in Figure 4. The overall distribution of the sum (left panel) follows a near-normal pattern, with the peak around mid-to-high-range values (4–6 out of 7), suggesting that most athletes exhibited a moderate-to-high number of correct descriptors. The distribution of the sum of descriptors by ‘grade’ (right panel) demonstrates that higher proficiency grades have higher sums of correct descriptors, supporting the relationship between the seven binary descriptor scores and the expert’s subjective overall grade.

The intra-rater reliability/agreement was substantial for all features, including weighted Cohen’s kappa: ‘grade’ (0.701); unweighted Cohen’s kappa: ‘foot’ (0.789), ‘lumbar’ (0.776), ‘knee’ (0.725), ‘depth’ (0.682), ‘pelvis/hip’ (0.662), and ‘trunk’ (0.637). Since ‘balance’ had no variability, as it was present in all the observations (see Figure 3 on descriptor prevalence), including when these were re-assessed, it was not included in the agreement analysis.

**Figure 5:**
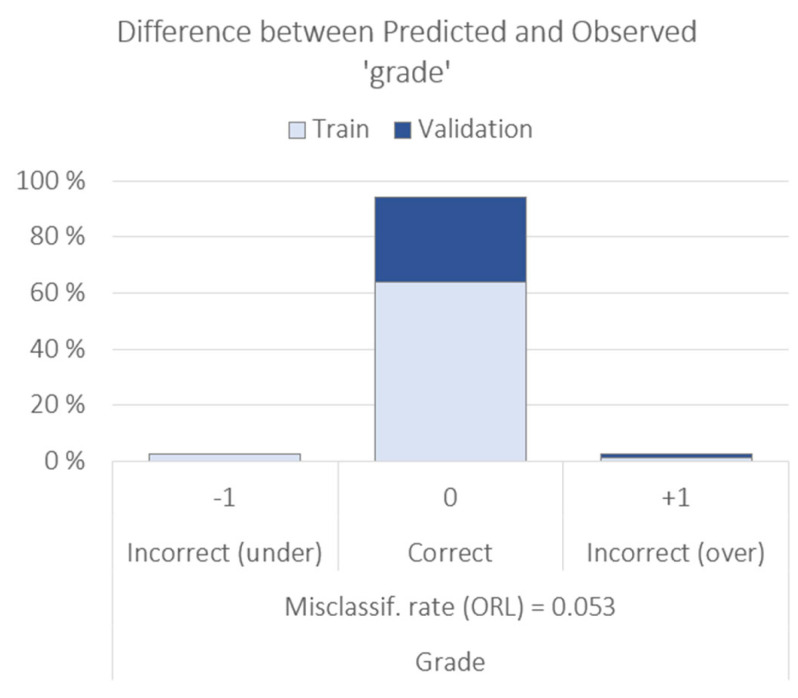
Decision tree misclassification rate for ‘grade’ using the subjective descriptors as input variables.

The model with the best/lowest misclassification rate for ‘grade’ (0.053 or 5.3%) was the ordinal logistic regression with subjective descriptors only as input variables, and stepwise was the variable selection method. The difference between the predicted and observed ‘grade’ is shown in Figure 5.

**Figure 6:**
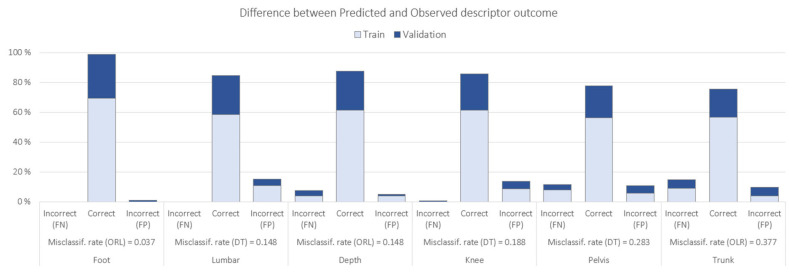
Misclassification rate of different models for each subjective descriptor using biomechanical data as input variables. FN = false negative. FP = false positive.

The models with the best/lowest misclassification rate for each descriptor as a target and the difference between predicted and observed ‘grade’ based on biomechanical data are shown in Figure 6.

## 4. Discussion

This study aimed to identify the extent to which subjective descriptors used by an expert can explain and be used to predict subjective overall ‘grades’ of single-leg squat proficiency. A secondary aim was to examine the ability to predict ‘grades’ and descriptors using the biomechanical data. To achieve the primary aim, a descriptive analysis and visualization was conducted to outline the prevalence of descriptors by ‘grade’, and two separate models (ORL and DT), with different settings, were fit to identify the relationship between the descriptors and the overall ‘grade’. To achieve the secondary aim, ORL and DT were also invoked to analyze the predictive capability of biomechanical data for each descriptor and the overall ‘grade’.

Most single-leg squats in our cohort were rated either 3 (fair) or 4 (good), with ‘balance’ invariably present across all observations. Substantial intra-rater agreement (weighted κ = 0.701 for ‘grade’; unweighted κ = 0.637–0.789 for ‘descriptors’) highlights consistency in the expert’s evaluations. Notably, the best predictive model for overall ‘grade’ (ORL’s misclassification rate: 5.3%) relied solely on the seven binary descriptors rather than on biomechanical measures, suggesting that these descriptors accurately mirror the expert’s thought process. The following sections delve into how these findings inform the interplay between subjective and objective assessments, as well as their practical integration in a real-world context to speed up and optimize the reliability of a movement proficiency assessment process.

The inspection of the descriptive statistics outlined in Figure 2 indicates that both the left and right ‘side’ present similar ‘grade’ distributions. In addition to this, the higher frequency for grade 4, ‘good’ movement proficiency, and the absence of grade 1, ‘very poor’ movement proficiency, is likely due to sample selection bias [38], as the subjects were the ones assessed by one sport organization (i.e., convenience sampling [39]). But, it could also be related to the assessment process itself [26] and the preconditioned assumptions or expected results by the rater [40], which would align with Pappalardo et al.’s research on subjective evaluation of human performance in sport [41]. As for the descriptor prevalence by ‘grade’ shown in Figure 3, the invariable presence of ‘balance’ in all grades stands out, indicating that this descriptor does not help in differentiating between grades. On the other hand, ‘pelvis/hip’, ‘knee’, and ‘trunk’ are the descriptors with lower presence in lower grades (i.e., less proficiency), suggesting that these three indicators may help the expert in identifying the worse single-leg squatters, as suggested by Nakagawa et al. [42] or Crossley et al. [43], in subjects with and without anterior knee pain. When analyzing the aggregation of correct descriptors (Figure 5), one can observe a positive relationship between this and the ‘grade’. The more ‘correct or yes’ descriptors in a given single-leg squat, the higher/better the movement proficiency ‘grade’ (see also the decision in Table 1).

**Figure 7:**
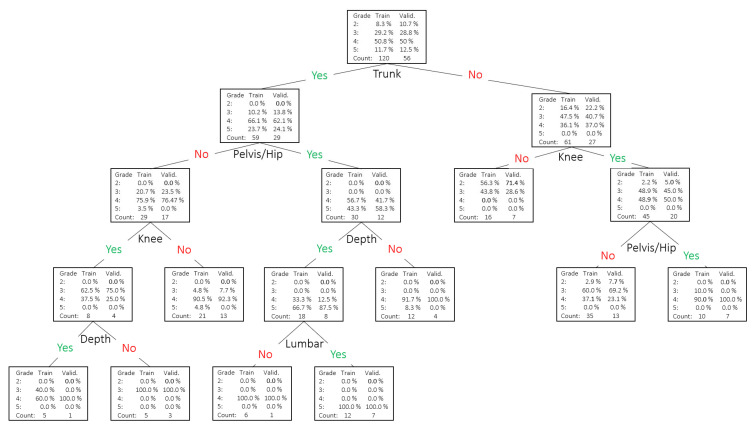
Decision tree for descriptors as inputs and ’grade’ as a target. A total of 87.8% accuracy (or a 12.2% misclassification rate).

The ordinal logistic regression provided an objective view of how descriptors of movement proficiency explain the overall ‘grade’, with a 94.7% accuracy (or 5.3% misclassification rate). In 2.7% of the cases, the prediction underestimated the ‘grade’ by 1 point (e.g., observed = 4; predicted = 3), and also, in 2.7% of the cases, it overestimated it (e.g., observed = 2; predicted = 3). The fact that there were no significant changes seen in the ORL output when analyzed separately by the ‘side’ indicates that the ‘grade’ given to a single-leg squat in the studied sample does not differ between the ‘side’, suggesting that laterality (or dominance [44]) seems to be not meaningful when predicting ‘grades’ based on the descriptors. The stepwise analysis removed ‘balance’ from the model, indicating no value for differentiating between ‘grade’, as also suggested by the descriptor prevalence analysis in Figure 2. The high accuracy by the ORL using the descriptors and the relationship between the aggregation of descriptors and ‘grade’ may indicate that the descriptor battery reflects well the expert’s thought process when assessing a single-leg squat. A decision tree, with an 87.8% accuracy, was invoked to provide a visual representation of how the expert tends to associate the descriptors with better or worse single-leg squat proficiency (Figure 7). In the DT, we observe that ‘grade’ (or single-leg squat proficiency) can be explained in various ways by various combinations of associated descriptors, but ‘pelvis/hip’, ‘knee’, ‘trunk’, and ‘depth’ are the descriptors with more explanatory worth.

The models using the kinematic and kinetic data did not improve the prediction accuracy for the ‘grade’ of the ORL with subjective descriptors only. This could indicate that the biomechanical features used cannot fully explain the movement proficiency assessment process. This may be a result of the descriptors of movement proficiency being influenced by the expert’s biases. Note that the intra-rater reliability for ‘grade’ was substantial (k = 0.701) [43], potentially because they consider other contextual information, such as joint coupling or multi-segment coordination [1]. Likewise, it has also been suggested that discrete descriptive statistics of temporal data such as mean, standard deviation, range, or values at max peak knee flexion, for instance, widely used in biomechanical research [45], are not enough to describe human movement coordination, thereby discarding much of the information contained in time-series data [46].

**Figure 8:**
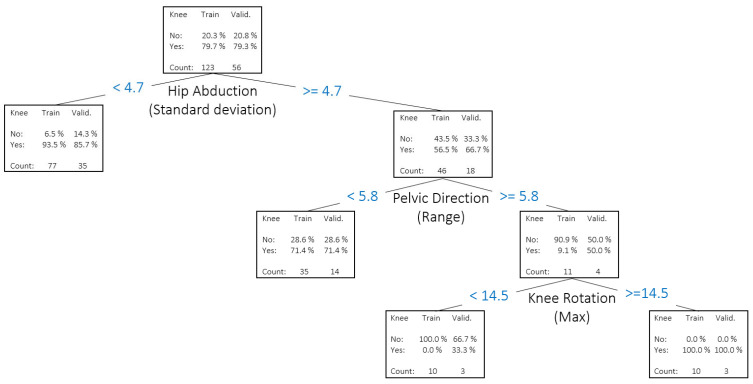
Decision tree for the descriptor ’knee’ and biomechanical data as input variables.

When modeling the different subjective descriptors as targets with the biomechanical data as inputs, ORL and DT models showed variable accuracy, depending on the descriptor (see Figure 6). The model for ‘foot’ was the most accurate one (96.3%), followed by ‘lumbar’ and ‘depth’ (both 85.2%), ‘knee’ (81.2%), ‘pelvis/hip’ (71.7%), and ‘trunk’ (62.3%). This order of accuracy follows almost the same order as the intra-rater agreement for descriptors: ‘foot’ (0.789), ‘lumbar’ (0.776), ‘knee’ (0.725), ‘depth’ (0.682), ‘pelvis/hip’ (0.662), and ‘trunk’ (0.637). This could indicate that ‘trunk’, ‘pelvis/hip’, and ‘depth’ are the hardest descriptors to assess by the expert. In this sense, previous research has reported some experts to have just moderate (k < 0.60) intra-rater reliability [43,47], suggesting that reliably assessing human movement proficiency is not an easy task. Predicting performance for descriptors such as ‘trunk’, pelvis/hip’, and ‘depth’ tends to be more challenging than for ‘foot’ or ‘knee’ due to their inherently complex, multi-segment nature. For instance, Ugalde et al. (2015) [7] demonstrated that visually evaluating pelvic tilt and trunk lean during single-leg activities leads to higher rater variability because these motions are subtle and easily influenced by total-body balance strategies. Munro et al. (2017) [5] similarly noted that while knee valgus can often be captured reliably using two-dimensional analysis, multi-plane trunk or hip deviations are less straightforward to quantify consistently. In addition, Crossley et al. (2011) [43] found that hip abductor muscle timing and lateral trunk control played a significant role in single-leg squat mechanics—two elements that are more difficult to assess visually without precise instrumentation. These findings highlight why ‘pelvis/hip’ and ‘trunk’ descriptors can exhibit greater inaccuracies in subjective rating. They require detecting small changes in frontal, sagittal, and sometimes transverse planes, all happening rapidly during a squat. Finally, Kivlan and Martin (2012) [6] emphasized how squat depth involves a coordinated effort across the ankles, knees, hips, and trunk. Any shortfall in one region—be it limited range of motion or delayed neuromuscular control—may manifest as compensations elsewhere, making ‘depth’ a tricky, composite variable to assess reliably. In short, these descriptors involve a more complex interplay of muscle groups and joint movements, leading to increased variability and lower predictive accuracy when compared to simpler, more focal descriptors, like ‘foot’ or ‘knee’. Hence, the use of objective biomechanical data involving richer time-series metrics (e.g., joint coupling or phase-based analyses) can assist the expert in some cases, defining objective thresholds to rate a descriptor, thus improving consistency and reliability. Likewise, the development of visual tools to explore the best explanatory biomechanical variables in relation to each descriptor may reinforce the understanding of the expert’s thought process [48,49]. See the decision tree for ‘knee’ in Figure 8 as an example. In contrast, the higher accuracy for descriptors, like ‘foot’ and ‘lumbar’, suggests that these features were relatively straightforward to quantify with our available biomechanical measures (e.g., clearly defined angular or positional data).

In an applied setting, these models could be implemented with three main goals: (1) to automatically recommend ‘grades’ based on the imputed subjective descriptors, increasing the speed and reliability of the assessment; (2) to reinforce and review the thought process of the evaluators (feedback), giving them references from the objective data that will improve future assessments; and (3) to advocate for performance evaluators (i.e., physiotherapists, athletic trainers, strength and conditioning coaches) to utilize both types of evaluations, subjective assessment, and biomechanical data and to be aware of their differences. Further, it also encourages the need for the experts to be aware of the various reasons that could account for these differences, as well as the tendencies of the subjective assessments. As an example, the objective measures, the ways that have been imputed in this study, may not capture and fully account for certain aspects of the single-leg squat, such as multi-segment coordination, which would be important to know when evaluating movement proficiency. Alternately, the subjective assessor may be prone to certain biases or incapable of reliably evaluating a three-dimensional movement involving several joints and segments and may consistently under- or over-rate certain subjects.

Some limitations of this study should be acknowledged. From a predictive modeling perspective, other models (e.g., neural networks or support vector machines) might have yielded higher accuracy; however, their ‘black-box’ nature limits interpretability, making them less suitable for this context. Additionally, this retrospective study was conducted on male athletes due to the structured training environment in which data collection took place, resulting in a convenience sample. While this ensured a controlled and homogenous dataset, and the proposed methodology is transferable to other contexts, it limits the generalizability of our findings, particularly given documented sex-based differences in movement patterns and neuromuscular control during single-leg squats, including knee valgus tendencies and hip muscle activation strategies [42,50,51]. Furthermore, all movement assessments were conducted by a single expert, which, while ensuring consistency, may introduce subjective bias. Aligning the model solely with one evaluator’s judgments could inadvertently reinforce inherent grading tendencies.

Future research should address these limitations by incorporating multiple evaluators, investigating how automated feedback influences rater bias, and expanding biomechanical analyses to include time-series metrics (e.g., joint coupling, cross-correlation, relative phases, or functional data analysis), potentially enhanced by deep learning approaches. As new objective data streams become available [9], integrating subjective assessments with automated measures has the potential to improve accuracy, efficiency, and reliability, leading to more robust, hybrid decision-making processes in movement proficiency evaluation [2,27].

## 5. Conclusions

The models developed in this study provide an explanation of the subjective assessment of single-leg squat proficiency. Specifically, they demonstrate that subjective perceptions of descriptors can be somewhat accurately explained through a small number of biomechanical indicators. While advancements in objective data collection and marker-less tracking systems continue to evolve, the results of this study reinforce the importance of combining both subjective and objective assessments for a comprehensive evaluation of an athlete’s movement proficiency.

Incorporating decision support systems and decision analysis methods—such as interpretable models and automated feedback—can enhance the evaluator’s practical workflow by highlighting key risk factors and movement errors more quickly. Rather than relying solely on subjective judgment, evaluators receive structured guidance (e.g., probabilistic insights or ‘if-then’ decision trees) that clarifies why certain squats or descriptors might warrant closer attention. This objective layer of analysis can streamline discussions among interdisciplinary teams (physiotherapists, strength coaches, etc.), reduce potential biases, and support more consistent, data-driven decisions in training or rehabilitation settings.

## Figures and Tables

**Table 1:** Example of a decision table for ‘grade’ 2, 3, 4, and 5. Note that ‘grade’ is separate from being the sum of the descriptors.

	Foot	Knee	Pelvis/Hip	Lumbar	Trunk	Balance	Depth	Grade
Subject 1	Yes	No	No	Yes	No	Yes	No	2
Subject 2	No	No	No	Yes	No	Yes	Yes	2
Subject 3	Yes	No	No	No	No	Yes	No	2
Subject 4	Yes	No	No	No	No	Yes	No	2
Subject 5	Yes	No	No	No	No	Yes	No	2
Subject 6	Yes	No	No	Yes	Yes	Yes	No	3
Subject 7	Yes	Yes	No	Yes	No	Yes	No	3
Subject 8	Yes	No	Yes	Yes	No	Yes	No	3
Subject 9	Yes	Yes	No	Yes	No	Yes	No	3
Subject 10	Yes	No	No	Yes	Yes	Yes	No	3
Subject 11	Yes	Yes	No	Yes	Yes	Yes	No	4
Subject 12	Yes	Yes	No	Yes	No	Yes	Yes	4
Subject 13	Yes	Yes	Yes	Yes	Yes	Yes	No	4
Subject 14	Yes	Yes	Yes	Yes	No	Yes	Yes	4
Subject 15	Yes	Yes	Yes	Yes	Yes	Yes	No	4
Subject 16	Yes	Yes	Yes	Yes	Yes	Yes	Yes	5
Subject 17	Yes	Yes	Yes	Yes	Yes	Yes	Yes	5
Subject 18	Yes	Yes	Yes	Yes	Yes	Yes	Yes	5
Subject 19	Yes	Yes	Yes	Yes	Yes	Yes	Yes	5
Subject 20	Yes	Yes	No	Yes	Yes	Yes	Yes	5

**Table 2:** Descriptive statistics for kinematic data. @PKF: value at peak knee flexion; SD: standard deviation.

Label	N	Average (Mean)	Pooled SD	Average (Min)	SD (Min)	Average (@PKF)	SD (@PKF)	Average (Max)	SD (Max)	Average (Range)	SD (Range)
Thoracic Flexion	176	30.0	5.4	20.3	12.4	36.0	6.5	36.5	14.7	16.2	6.1
Thoracic Ipsilateral Rotation	176	2.3	1.7	−1.7	3.7	3.7	10.9	4.4	4.1	6.1	8.7
Thoracic Ipsilateral Flexion	176	7.3	4.1	1.5	6.5	9.0	4.9	10.2	7.2	8.7	13.6
Pelvic Tilt	176	6.4	5.1	−3.8	10.9	9.3	5.3	9.8	14.3	13.6	5.8
Pelvic Direction	176	−0.8	2.2	−3.0	4.9	−0.3	11.7	2.9	4.5	5.8	6.3
Pelvic Ipsilateral Side Bend	176	−1.2	2.4	−2.9	5.3	−2.2	5.8	3.5	4.6	6.3	42.5
Standing Hip Flexion	176	27.5	14.1	−0.3	11.7	42.0	10.6	42.2	17.0	42.5	8.2
Standing Hip Rotation	176	6.8	2.6	2.6	5.8	8.8	4.9	10.8	6.5	8.2	13.7
Standing Hip Abduction	176	−10.2	5.9	−15.2	10.6	−14.2	5.7	−1.5	5.6	13.7	47.5
Standing Knee Flexion	176	41.4	14.5	12.3	4.9	59.8	3.3	59.8	9.9	47.5	8.3
Standing Knee Rotation	176	2.9	2.4	−0.9	5.7	1.7	3.8	7.5	5.5	8.3	7.4
Standing Knee Abduction	176	−4.3	2.4	−8.7	5.3	−7.7	6.0	−1.4	4.2	7.4	18.7
Standing Ankle Flexion	176	2.8	5.5	−8.8	3.8	9.4	3.1	9.8	5.7	18.7	11.2
Standing Ankle Abduction	176	19.0	3.3	11.3	6.0	20.4	14.7	22.5	6.5	11.2	5.1
Standing Ankle Inversion	176	−2.5	1.4	−5.7	3.1	−4.0	4.1	−0.6	3.1	5.1	182.2

## Data Availability

The data presented in this study are available on request from the corresponding author. The data are not publicly available due to institutional data privacy policies.

## Abbreviations

The following abbreviations are used in this manuscript:

DT	Decision Tree
EM	Enterprise Miner
MP	Movement Proficiency
ORL	Ordinal Logistic Regression

## References

[1] McIntosh S., Kovalchik S., Robertson S. (2019). Comparing subjective and objective evaluations of player performance in Australian Rules football. PLoS ONE.

[2] Sieghartsleitner R., Zuber C., Zibung M., Conzelmann A. (2019). Science or Coaches’ Eye?—Both! Beneficial Collaboration of Multidimensional Measurements and Coach Assessments for Efficient Talent Selection in Elite Youth Football. J. Sports Sci. Med..

[3] Roberts A.H., Greenwood D.A., Stanley M., Humberstone C., Iredale F., Raynor A. (2019). Coach knowledge in talent identification: A systematic review and meta-synthesis. J. Sci. Med. Sport.

[4] Schelling X., Robertson S. (2020). A development framework for decision support systems in high-performance sport. Int. J. Comput. Sci. Sport.

[5] Munro A., Herrington L., Comfort P. (2017). The Relationship Between 2-Dimensional Knee-Valgus Angles During Single-Leg Squat, Single-Leg-Land, and Drop-Jump Screening Tests. J. Sport. Rehabil..

[6] Kivlan B.R., Martin R.L. (2012). Functional performance testing of the hip in athletes: A systematic review for reliability and validity. Int. J. Sports Phys. Ther..

[7] Ugalde V., Brockman C., Bailowitz Z., Pollard C.D. (2015). Single leg squat test and its relationship to dynamic knee valgus and injury risk screening. Pm R.

[8] Richter C., King E., Strike S., Franklyn-Miller A. (2018). Analyzing Human Movements—Introducing A Framework To Extract And Evaluate Biomechanical Data. bioRxiv.

[9] Hood S., McBain T., Portas M., Spears I. (2012). Measurement in Sports Biomechanics. Meas. + Control..

[10] Montull L., Slapšinskaitė-Dackevičienė A., Kiely J., Hristovski R., Balagué N. (2022). Integrative Proposals of Sports Monitoring: Subjective Outperforms Objective Monitoring. Sports Med.—Open.

[11] Rugai J. (2015). Methods of Biomechanical Analyses in Sports. Int. J. Second. Educ..

[12] Bennett H., Davison K., Arnold J., Martin M., Wood S., Norton K. (2019). Reliability of a movement quality assessment tool to guide exercise prescription (MOVEMENTSCREEN). Int. J. Sports Phys. Ther..

[13] Moran R.W., Schneiders A.G., Mason J., Sullivan S.J. (2017). Do Functional Movement Screen (FMS) composite scores predict subsequent injury? A systematic review with meta-analysis. Br. J. Sports Med..

[14] Bahr R. (2016). Why screening tests to predict injury do not work-and probably never will…: A critical review. Br. J. Sports Med..

[15] Hewett T.E., Myer G.D., Ford K.R., Heidt Jr R.S., Colosimo A.J., McLean S.G., Van den Bogert A.J., Paterno M.V., Succop P. (2005). Biomechanical measures of neuromuscular control and valgus loading of the knee predict anterior cruciate ligament injury risk in female athletes: A prospective study. Am. J. Sports Med..

[16] Krosshaug T., Steffen K., Kristianslund E., Nilstad A., Mok K.M., Myklebust G., Andersen T.E., Holme I., Engebretsen L., Bahr R. (2016). The Vertical Drop Jump Is a Poor Screening Test for ACL Injuries in Female Elite Soccer and Handball Players: A Prospective Cohort Study of 710 Athletes. Am. J. Sports Med..

[17] Yeadon M.R., Challis J.H. (1994). The future of performance-related sports biomechanics research. J. Sports Sci..

[18] Arlotti J.S., Carroll W.O., Afifi Y., Talegaonkar P., Albuquerque L., Ball J.E., Chander H., Petway A. (2022). Benefits of IMU-based Wearables in Sports Medicine: Narrative Review. Int. J. Kinesiol. Sports Sci..

[19] Simon H.A. (1978). Rational decision-making in business organizations. Nobel Memorial Lecture.

[20] Tversky A., Kahneman D. (1974). Judgment under uncertainty: Heuristics and biases. Science.

[21] Mørtvedt A.I., Krosshaug T., Bahr R., Petushek E. (2019). I spy with my little eye … a knee about to go ‘pop’? Can coaches and sports medicine professionals predict who is at greater risk of ACL rupture?. Br. J. Sports Med..

[22] Nevill A.M., Hemingway A., Greaves R., Dallaway A., Devonport T.J. (2017). Inconsistency of decision-making, the Achilles heel of referees. J. Sports Sci..

[23] Hill M., Scott S., Malina R.M., McGee D., Cumming S.P. (2019). Relative age and maturation selection biases in academy football. Science and Football.

[24] Damisch L., Mussweiler T. (2009). On the relativity of athletic performance: A comparison perspective on performance judgments in sports. Prog. Brain Res..

[25] Hodgson R.T. (2008). An Examination of Judge Reliability at a major U.S. Wine Competition. J. Wine Econ..

[26] O’Connor K., Cheema A. (2018). Do Evaluations Rise With Experience?. Psychol. Sci..

[27] Moradi M., Moradi M., Bayat F., Toosi A.N. (2019). Collective hybrid intelligence: Towards a conceptual framework. Int. J. Crowd Sci..

[28] Harriss D.J., Atkinson G. (2015). Ethical Standards in Sport and Exercise Science Research: 2016 Update. Int. J. Sports Med..

[29] Innovative Sports Training, Inc. (2017). The MotionMonitor Subject Setup using Digitization Method. https://www.youtube.com/watch?v=UuRYLsGz8Hs.

[30] Sinclair J., Taylor P.J., Hobbs S.J. (2013). Digital filtering of three-dimensional lower extremity kinematics: An assessment. J. Hum. Kinet..

[31] Khuu A., Foch E., Lewis C.L. (2016). Not all single leg squats are equal: A biomechanical comparison of three variations. Int. J. Sports Phys. Ther..

[32] Cohen J. (1968). Weighted kappa: Nominal scale agreement with provision for scaled disagreement or partial credit. Psychol. Bull..

[33] Cohen J. (1960). A Coefficient of Agreement for Nominal Scales. Educ. Psychol. Meas..

[34] Landis J.R., Koch G.G. (1977). The measurement of observer agreement for categorical data. Biometrics.

[35] Twala B. (2009). An empirical comparison of techniques for handeling incomplete data using decision trees. Appl. Artif. Intell..

[36] Bozdogan H. (1987). Model selection and Akaike’s Information Criterion (AIC): The general theory and its analytical extensions. Psychometrika.

[37] Ribeiro M.T., Singh S., Guestrin C. Why should i trust you?: Explaining the predictions of any classifier. Proceedings of the 22nd ACM SIGKDD International Conference on Knowledge Discovery and Data Mining.

[38] Tripepi G., Jager K.J., Dekker F.W., Zoccali C. (2010). Selection bias and information bias in clinical research. Nephron Clin. Pract..

[39] Bornstein M.H., Jager J., Putnick D.L. (2013). Sampling in Developmental Science: Situations, Shortcomings, Solutions, and Standards. Dev. Rev. DR.

[40] Teovanović P. (2019). Individual Differences in Anchoring Effect: Evidence for the Role of Insufficient Adjustment. Eur. J. Psychol..

[41] Pappalardo L., Cintia P., Pedreschi D., Giannotti F., Barabasi A.L. (2017). Human Perception of Performance. arXiv.

[42] Nakagawa T.H., Moriya É.T., Maciel C.D., Serrão F.V. (2012). Trunk, pelvis, hip, and knee kinematics, hip strength, and gluteal muscle activation during a single-leg squat in males and females with and without patellofemoral pain syndrome. J. Orthop. Sports Phys. Ther..

[43] Crossley K.M., Zhang W.J., Schache A.G., Bryant A., Cowan S.M. (2011). Performance on the single-leg squat task indicates hip abductor muscle function. Am. J. Sports Med..

[44] Whiteside D., Buszard T., Giblin G., Reid M., Loffing F., Hagemann N., Strauss B., MacMahon C. (2016). Skill acquisition in left- and right-handed athletes: Insights from elite coaching. Laterality in Sports: Theories and Applications.

[45] Lara O.D., Labrador M.A. (2013). A Survey on Human Activity Recognition using Wearable Sensors. IEEE Commun. Surv. Tutor..

[46] Lamb P.F., Bartlett R.M., Payton C.J., Burden A. (2018). Assessing Movement Coordination, in Biomechanical Evaluation of Movement in Sport and Exercise.

[47] Örtqvist M., Moström E.B., Roos E.M., Lundell P., Janarv P.M., Werner S., Broström E.W. (2011). Reliability and reference values of two clinical measurements of dynamic and static knee position in healthy children. Knee Surg. Sports Traumatol. Arthrosc..

[48] Valatavičius A., Gudas S. (2017). Towards the deep, knowledge-based interoperability of applications. Inf. Moksl..

[49] Sanders N.R., Manrodt K.B. (2003). Forecasting software in practice: Use, satisfaction, and performance. Interfaces.

[50] Zeller B.L., McCrory J.L., Kibler W.B., Uhl T.L. (2003). Differences in Kinematics and Electromyographic Activity between Men and Women during the Single-Legged Squat. Am. J. Sports Med..

[51] Willson J.D., Ireland M.L., Davis I. (2006). Core Strength and Lower Extremity Alignment during Single Leg Squats. Med. Sci. Sports Exerc..

